# African Swine Fever Epidemic, Poland, 2014–2015

**DOI:** 10.3201/eid2207.151708

**Published:** 2016-07

**Authors:** Krzysztof Śmietanka, Grzegorz Woźniakowski, Edyta Kozak, Krzysztof Niemczuk, Magdalena Frączyk, Łukasz Bocian, Andrzej Kowalczyk, Zygmunt Pejsak

**Affiliations:** National Veterinary Research Institute, Puławy, Poland

**Keywords:** African swine fever, wild boar, pigs, epidemiology, epidemic, viruses, Poland

## Abstract

In Poland, African swine fever (ASF) emerged in February 2014; by August 2015, the virus had been detected in >130 wild boar and in pigs in 3 backyard holdings. We evaluated ASF spread in Poland during these 18 months. Phylogenetic analysis indicated repeated incursions of genetically distinct ASF viruses of genotype II; the number of cases positively correlated wild boar density; and disease spread was very slow. More cases were reported during summer than autumn. The 18-month prevalence of ASF in areas under various animal movement restrictions was 18.6% among wild boar found dead or killed by vehicles and only 0.2% in hunted wild boar. Repeated introductions of the virus into the country, the primary role of wild boar in virus maintenance, and the slow spread of the disease indicate a need for enhanced biosecurity at pig holdings and continuous and intensive surveillance for fast detection of ASF.

## Abstract 2

Epidemiologic and phylogenetic analyses suggest that the virus was repeatedly introduced and that the disease is maintained in wild boar.

African swine fever (ASF) is an infectious and notifiable disease of domestic and wild animals of the family Suidae (*1**,**2*). First described in Kenya in 1921, ASF was territorially restricted to Africa only until 1957, when it spread from Angola to Lisbon. From then on, ASF has been repeatedly detected in many countries of Europe, Central America, and South America. In some countries (e.g., France, Belgium, the Netherlands), ASF outbreaks were rapidly contained, but in others (e.g., Portugal and Spain) ASF virus (ASFV) persisted for >30 years. Another long-time infected region in Europe is Sardinia (Italy), where ASFV has been circulating since 1978 and where the disease has been maintained as endemic (*3*). In 2007, the most recent epidemic started in Georgia and thereafter moved to Armenia, Azerbaijan, and the Russian Federation (*4**,**5*). In 2012 and 2013, ASF occurred in Ukraine and Belarus, respectively, and in 2014, it crossed into the European Union. According to the World Organisation for Animal Health, >550 ASF cases among wild boar and outbreaks among domestic pigs were detected through 2015 in Estonia, Latvia, Lithuania, and Poland (*5*).

In Poland, the first cases of ASF were detected in wild boar in February 2014 in the northeastern part of the country, very near (<1 km) the border with Belarus (*6*). As of August 31, 2015, a total of 76 cases in wild boar and 3 outbreaks among domestic pigs had been found in 3 counties (basic administrative regions of Poland). 

Extensive surveillance revealed a unique pattern of disease spread that did not fit the commonly perceived concept of ASF epidemiology. Our study objective was to describe the spatiotemporal spread of ASF in Poland during the first 18 months after detection of the first cases.

## Materials and Methods

### Surveillance Design and Diagnostic Tests

After the first cases of ASF in Poland were confirmed, the affected area was differentiated into 3 levels of risk: area I (regions free from ASF but located near areas where ASF had been occurring in wild boar), area II (ASF detected in wild boar only), and area III (established after detection of ASF in pigs) (*7**,**8*). Despite differences with regard to animal movement restrictions, the surveillance strategy applied to areas I–III was the same: all wild boar found dead and those killed in road accidents (passive surveillance) and hunted wild boar (active surveillance) from all areas were submitted for testing. Samples collected from dead wild boar were whole blood, serum, marrow bones, kidneys, liver, spleen, lymph nodes, and lungs; samples from hunted wild boar were whole blood and serum. Homogenates (10% wt/vol) of individual tissues were prepared in phosphate-buffered saline. Clarified material was stored at −80°C or directly used for virus DNA extraction. Virus DNA was extracted directly from 200-μL aliquots of serum or tissue sample homogenates by using the commercial QIAamp DNA Mini Kit (QIAGEN, Hilden, Germany) according to the manufacturer’s recommended procedures. We used a PCR with the ASF diagnosis primers and a commercial probe (Universal ProbeLibrary no. 162; Roche Applied Science, Branford, CT, USA), which generates an amplicon of 74 bp within viral protein 72, to confirm the presence of ASFV DNA. Specific primers and probes were added to a LightCycler 480 Probes Master Kit (Roche Applied Science), and reactions were performed in a Stratagene Mx3005P real-time PCR thermocycler (Agilent Technologies, Santa Clara, CA, USA) according to the protocol described by Fernández -Pinero et al. (*9*).

Altogether, from February 2014 through August 2015, samples from 609 dead/road accident wild boar and 12,253 hunted wild boar from areas I–III (*7**,**8*), as well as from ≈35,000 domestic pigs, were tested by real-time PCR; detailed results and difficulties encountered during the diagnostic process are described elsewhere (*10*). According to terminology adopted in Poland, outbreaks were defined as the detection of DNA of ASFV in pigs (irrespective of the number of pigs in a holding), and cases were defined as the presence of viral DNA in >1 wild boar found at the same time and in the same place. Thus, the number of infected animals outnumbered the number of cases or outbreaks. However, for the purpose of prevalence calculations, we took into account individual animals. To calculate the annual prevalence of ASF in wild boar during the first year of the epidemic and to analyze potential seasonal variations, we established prevalence rates (with 95% CIs) separately for wild boar tested within the scope of active and passive surveillance in quarterly intervals: spring (March–May 2014), summer (June–August 2014), autumn (September–November 2014), and winter (December 2014–February 2015). In addition, we calculated prevalence in monthly intervals to encompass the period from the beginning of the epidemic in February 2014 through August 2015. We mapped the locations of ASF outbreaks and cases by using sampling location coordinates in ArcGIS for Desktop software (Esri Inc., Redlands, CA, USA). 

### DNA Sequencing and Phylogenetic Analysis

We used the DNA of ASFV representing 64 cases and 3 outbreaks for phylogenetic analysis. So far we have failed to produce proper-length readable sequences for samples from case nos. 20, 24, 26–28, 32, 51, 56, 57, and 68. The primers specific to the MGF505-2R gene ASFV sequence were designed on the basis of the complete genome sequence of the BA71V strain (GenBank accession no. U18466.2) by using online Primer 3 Plus software (http://www.bioinformatics.nl/primer3plus/). The primers were also 100% complementary to the Georgia 2007/1 sequence strain. The expected product length was estimated to be 1,173 bp. The primer sequences used for amplification and sequencing of the MGF505–2R fragment were LVR13F: 5′-GCAGAGGTATGATGTCCTTA-3′ and LVR13F: 5′-TTCCTGTTGAACAAGTATCT-3′. The PCR products were separated in a 1.5% agarose gel (Invitrogen, Grand Island, NY, USA) and then purified according to the procedure for the QIAquick Gel Extraction Kit (QIAGEN). The amplicons were sequenced on a GS FLX/Titanium sequencer (Roche Applied Science) by Centrum Badań DNA Service (Poznań, Poland). Each product was sequenced in forward and backward directions and then assembled into a single contig by using Geneious R7 software (Biomatters Ltd., Auckland, New Zealand). The ClustalW alignment calculation parameters in MEGA6 (*11*) were as follows: gap opening penalty 15, gap extension penalty 6.66, transition weight 0.5, and delay divergent cutoff 30%. We plotted the phylogram by using the neighbor-joining algorithm in MEGA6 software and calculated the nucleotide similarity matrix providing the information about the sequence identity by using Geneious R7 software. The obtained nucleotide sequences of ASFV isolates were trimmed, assembled into contigs, and aligned by using Geneious R7 software. We also retrieved 2 sequences of ASFV representing genotype II (Georgia 2007/1 and Odintsovo/2014 Russia) from GenBank to use for comparison. The tree was rooted against ASFV strains Warmbaths South Africa and Malawi Lil 20/1, representing genotypes IV and VIII, respectively. We submitted the nucleotide sequences of ASFV successfully sequenced in Poland to GenBank under accession nos. KT366447–KT366459 and KT900042–KT900107.

### Statistical Analyses

To evaluate correlations between the number of ASFV-positive wild boar and wild boar density in the forestry units in which ASF detections were notified during the first year after the beginning of the epidemic, we performed a Pearson and Spearman correlation analysis (significance level 0.05). The follow-up analysis was performed 3 months after detection of ASF in new areas, which led to enlargement of the infected zone in August 2015. To assess statistical differences between seasonal prevalence of ASF, we used the Fisher exact test with a Bonferroni correction for each single comparison (significance level 0.05).

## Results

**Figure 1:**
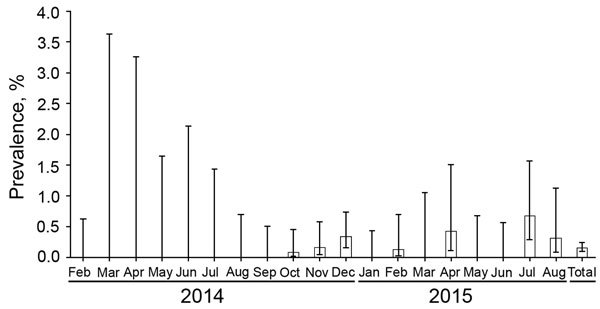
Monthly prevalence of African swine fever in hunted wild boar, Poland, February 2014–August 2015. Error bars indicate 95% CIs.

**Figure 2:**
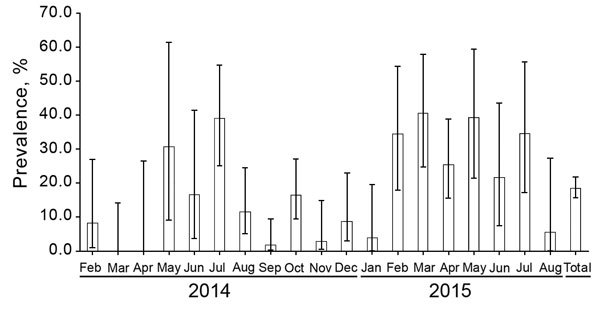
Monthly prevalence of African swine fever in dead (including road accident deaths) wild boar, Poland, February 2014–August 2015. Error bars indicate 95% CIs.

The average annual prevalence of ASFV (based on positive PCRs) among hunted wild boar was 0.12% (95% CI 0.1%–0.2%) (Table). Prevalence did not differ significantly by season. With regard to detection of ASFV in dead wild boar, the annual prevalence was 14.2% (95% CI 11.1%–17.9%) and ranged from 8.2% in spring to 24.3% in summer. The only significant difference (after taking the Bonferroni correction into account) was between summer and autumn (p<0.001). The monthly prevalence ranged from 0 to 0.7% among hunted wild boar and from 0 to 40.5% among dead wild boar (Figures 1, 2). The overall 18-month prevalence in areas under animal movement restrictions was 18.6% (95% CI 15.7%–21.8%) according to passive surveillance and 0.2% (95% CI 0.1%–0.2%) according to active surveillance.

**Table:** African swine fever in wild boar in Poland during the first year after detection of the first cases in February 2014

Season	Active surveillance*					Passive surveillance†			
	No. positive	No. negative	Total	Prevalence, % (95% CI)		No. positive	No. negative	Total	Prevalence, % (95% CI)
Spring	0	446	446	0 (0–0.9)		4	45	49	8.2 (3.2–19.2)
Summer	0	988	988	0 (0–0.4)		26	81	107	24.3 (17.2–33.2)
Autumn	3	3,270	3,273	0.09 (0–0.3)		13	144	157	8.3 (4.913.7)
Winter	7	3,453	3,460	0.2 (0.1–0.4)		14	75	89	15.7 (9.6–24.7)
Total	10	8,157	8,167	0.12 (0.1–0.2)		57	345	402	14.2 (11.1–17.9)

*Hunted. †Found dead.

**Figure 3:**
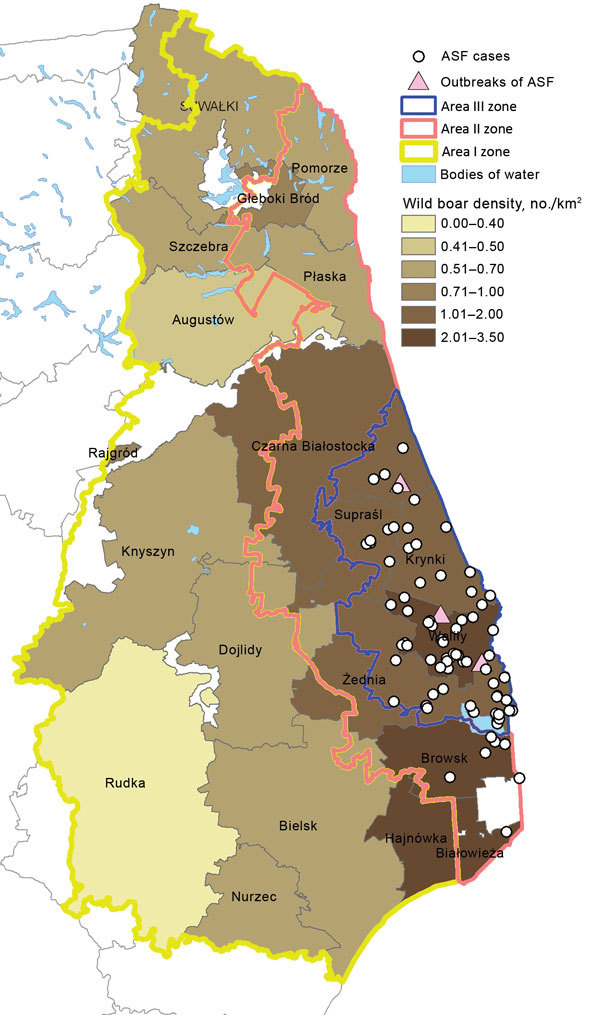
Locations of African swine fever (ASF) cases and outbreaks in Poland. Wild boar density data based on the National Forestry Service of Poland census.

We found a correlation between the number of ASF notifications and the number of wild boar in the affected forestry units (Spearman rank correlation coefficient R = 0.90, p<0.05) in February 2015 (during the first year after detection of the first case). As of August 2015 (after detection of ASF in new areas in June 2015 and the enlargement of the infected zone), the correlation lost statistical significance (Figure 3).

**Figure 4:**
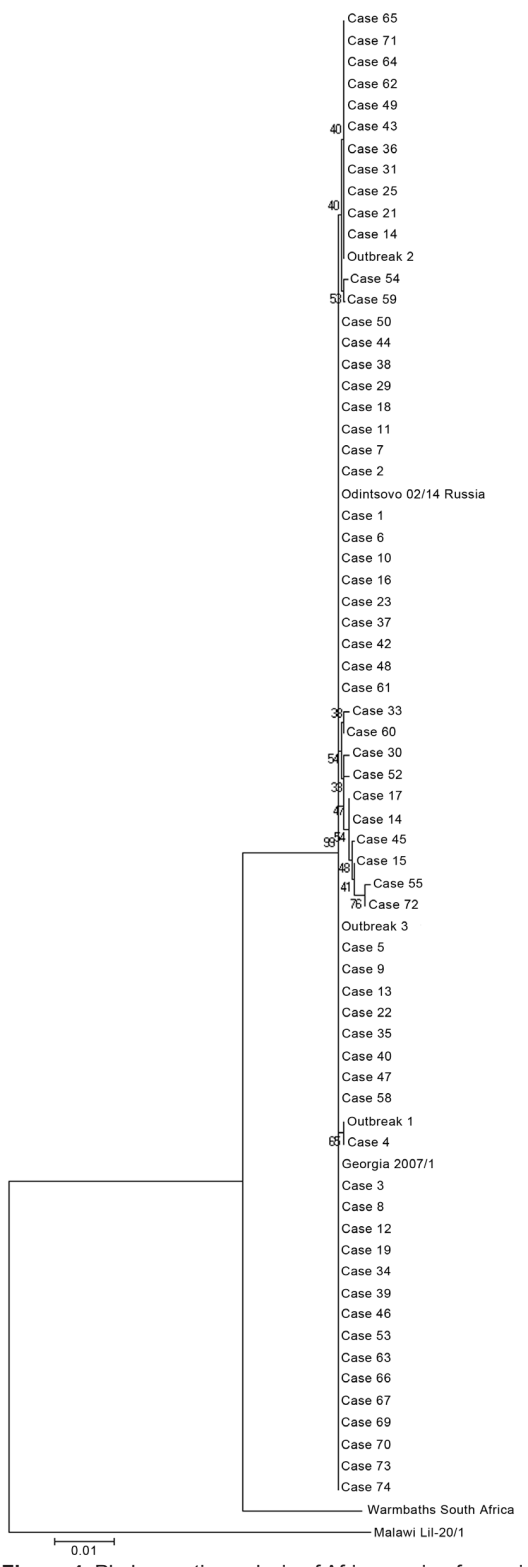
Phylogenetic analysis of African swine fever virus detected in pigs (outbreaks) and wild boar (cases) in Poland. Numbers on branches indicate bootstrap coefficient values. Scale bar indicates nucleotide substitutions per residue.

**Figure 5:**
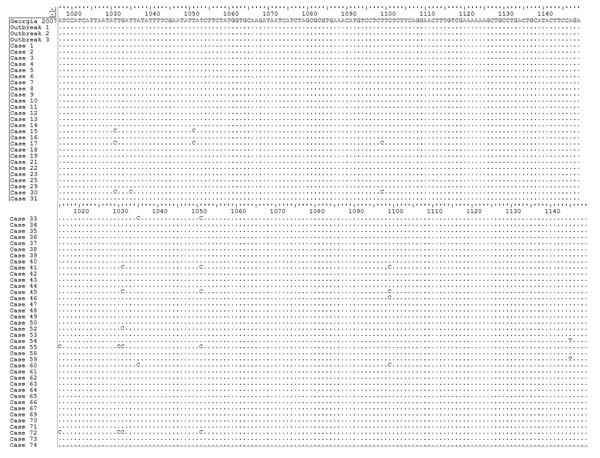
Nucleotide alignment of the MGF505–2R gene variable sequence fragment (residues from 1,015 to 1,149 nt) showing point mutations and differences between isolate Georgia 2007/1 isolate and African swine fever virus field isolates from Poland. The graph was generated by using Bioedit version 7.2.5 software (Ibis Biosciences, Carlsbad, CA, USA). The dots indicate identical nucleotide residues. The variable residues are visible as a nucleotide symbol.

The nucleotide and amino acid sequence identity of the MGF505-2R gene between ASFV isolates from Poland ranged from 99.47% to 100%. The largest cluster consisted of 42 sequences (41 from wild boar and 1 from pigs [outbreak 3]) exhibiting 100% homology between each other and indistinguishable from 2 references included for comparison: Georgia 2007/1 and Odintsovo 02/14 Russia (Figure 4). The second largest group containing 100% homologous sequences comprised 12 viruses (11 from wild boar and 1 from pigs [outbreak 2]) with 99.9% similarity to viruses of the previous group. The DNA fragment of the virus recovered from pigs identified as from the first outbreak differed slightly from those mentioned above, and the only identical sequence was from the virus from case no. 4. Sequences representing case nos. 15, 17, 41, 45, 55, and 72 formed a clearly separate and diverse cluster (within-group genetic diversity 99.5%–99.9%) (Figure 5).

## Discussion

After the emergence of ASF in Poland, the preliminary forecasts had predicted that the virus would deplete the population of wild boar in the region or would spread quickly to new areas because it is inherently so highly contagious. These predicted events, however, did not occur. Nor did the concept that ASFV cannot be sustained among wild boar without spillover from domestic pigs (*12**,**13*) apply to the situation in Poland. So far, the number of cases in wild boar in Poland has greatly outnumbered outbreaks among domestic pigs. The virus has been found almost exclusively in wild boar, which seem to be the sole mediator for virus dissemination. The total area of the infected region is only ≈1,500 km^2^. The slow spatial spread of ASF may be associated with the social behavior of wild boar, which has been studied quite extensively in Białowieża Primeval Forest, straddling the Poland–Belarus border (*14*). Wild boar show strong site fidelity, and most (≈70%) stay within 1–2 km of the center of their natal home ranges; only a relatively small percentage (5%–10%) of the population disperses from their natal range but not farther than 20–30 km. Spatial overlap of family groups is limited (*15*), which hampers transmission of the virus between groups by either direct contact between susceptible and sick animals or indirect contact with infected carcasses. In addition, the high virulence of the virus, which leads to the high case-fatality rate, prevents infected wild boar from long-distance movements. Therefore, long-distance dispersal of the virus by wild boar as carriers is assumed to be unlikely and mostly requires human involvement. However, specific socio-agricultural conditions in the affected region (i.e., low pig density, very few commercial farms, and small-scale national and international trade) create favorable barriers hitherto preventing the spread of the virus over long distances. It seems that the overall effect on the population was not significant and that, despite a high lethality rate of genotype II for wild boar (no. deaths/no. infected animals) (*16**,**17*), the mortality rate (no. deaths/no. animals in the affected population) seems to not be very high. Therefore, the virus does not seem to be highly contagious, which can also be explained on one side by the inherent epidemiologic properties of ASF (no airborne transmission and required contact with blood or excretions of infected animals) and on the other side from the specific behavior of wild boar described above. The results obtained in our study provide grounds for redefining the role of wild boar, which after 18-months of observation can be considered as a reservoir host for ASFV.

The complete genetic identity between a large cluster of Poland ASFV isolates with Georgia 2007/1 isolates clearly shows that the examined region, although relatively variable, can remain highly conservative for a long time (8 years). On the other hand, the genetic divergence of up to 0.5% in viruses from Poland highlighted by the presence of separate clusters on the phylogenetic tree clearly indicates that Poland has experienced a few incursions of genetically distinct ASFVs of genotype II. This finding is also supported by epidemiologic observations: 29 of 76 cases were located no farther than 5 km from the border with Belarus. With respect to outbreaks among pigs, the phylogenetic analysis clearly indicates no direct link between the 3 outbreaks. Epidemiologic investigations showed that wild boar were the most likely source of infection for domestic pigs (mainly poor biosecurity of pig holdings, enabling contact with wild animals). Overall, results of phylogenetic studies demonstrate the dynamic nature of the ASF epidemic in eastern Europe and raise serious concerns for control of ASF. We emphasize that without close and transparent collaboration between ASF-affected countries, eradication goals will be difficult to achieve.

The statistical relationship between wild boar density and the number of ASF cases was found after the 12 months after the beginning of the epidemic. The correlation was not statistically significant a few months after the virus spread to new forestry units with high wild boar density, apparently because of substantial changes in the population size in areas II and III as a result of introduced control measures (according to the most current census, the population in the aforementioned areas decreased by ≈25%). Moreover, the analysis of combined data for Poland and the Baltic States, conducted by a panel of European Food Safety Authority experts, found no correlation between wild boar density and ASF case notifications (*18*). This issue requires clarification, and the analysis will be continuously updated. However, during the first year, all cases in wild boar were detected in areas with a wild boar density of >1 animal/km^2^ (Figure 3); currently, in areas where substantial efforts have been undertaken to reduce wild boar populations, the number of ASF notifications has been reduced considerably. This finding raises potential implications for ASF control strategies (i.e., maintaining the wild boar population in the affected region at the level of ≈0.5–0.7 animals/km^2^) and can be taken into account as a control option for reducing the number of cases among wild boar. Moreover, maintaining the population density in the surrounding regions at a low level may create a low-density barrier, preventing the virus from becoming established among wild boar in new areas, and infections, if they occur, can be expected to die out. As indicated in the most recent European Food Safety Authority report (*18*), the low density of wild boar can be achieved by female-targeted boar hunting and a feeding ban. However, this approach should be applied as a long-term control measure because intensive hunting is logistically demanding. It would also be desirable to significantly reduce the domestic pig population from backyard pig holdings, which do not fulfill biosecurity requirements. This process would thus create a chance to minimize the major risk for long-distance dispersal of the virus, which is attributed to human activity (e.g., transfer of contaminated pork, pig waste, or fomites to other, sometimes remote, regions). A new biosecurity regulation is being put in place in areas II and III, which, among other things, stipulates that holdings that do not fulfill the strict requirements will be closed.

In summary, during 18 months of ASF in Poland, we observed repeated introductions of ASFV into the country, slow spread of the disease in areas of dense wild boar populations, and a primary role of wild boar in virus maintenance. Enhancement of biosecurity practices at pig holdings is crucial for minimizing the risk for virus spillover virus from wild to domestic populations followed by long-distance spread of ASFV by human-related activities. Continuous and intensive surveillance enabling fast detection of ASF is needed, especially in previously disease-free areas.
